# Prediction model for the pretreatment evaluation of mortality risk in anti-melanoma differentiation-associated gene 5 antibody-positive dermatomyositis with interstitial lung disease

**DOI:** 10.3389/fimmu.2022.978708

**Published:** 2022-09-23

**Authors:** Xianhua Gui, Wangzhong Li, Yanzhe Yu, Tingting Zhao, Ziyi Jin, Kaifang Meng, Rujia Wang, Shenyun Shi, Min Yu, Miao Ma, Lulu Chen, Wei Luan, Xiaoyan Xin, Yuying Qiu, Xiaohua Qiu, Yingwei Zhang, Min Cao, Mengshu Cao, Jinghong Dai, Hourong Cai, Mei Huang, Yonglong Xiao

**Affiliations:** ^1^ Department of Respiratory Medicine, The Affiliated Drum Tower Hospital of Nanjing University Medical School, Nanjing, China; ^2^ Department of Thoracic Surgery/Oncology, China State Key Laboratory of Respiratory Disease & National Clinical Research Center for Respiratory Disease, The First Affiliated Hospital of Guangzhou Medical University, Guangzhou, China; ^3^ Department of Nasopharyngeal Carcinoma, Sun Yat-sen University Cancer Center, The State Key Laboratory of Oncology in South China, Collaborative Innovation Center for Cancer Medicine, Guangzhou, China; ^4^ Guangdong Key Laboratory of Nasopharyngeal Carcinoma Diagnosis and Therapy, Sun Yat-Sen University Cancer Center, Guangzhou, China; ^5^ Department of Rheumatology and Immunology, The Affiliated Drum Tower Hospital of Nanjing University Medical School, Nanjing, China; ^6^ Department of Radiology, The Affiliated Drum Tower Hospital of Nanjing University Medical School, Nanjing, China

**Keywords:** anti-melanoma differentiation-associated gene 5 antibody-positive dermatomyositis with interstitial lung disease, cytokeratin 19 fragment, anti-Ro52 antibody, risk score, prediction model

## Abstract

**Background:**

Anti-melanoma differentiation-associated gene 5 antibody-positive dermatomyositis with interstitial lung disease (anti-MDA5 DM-ILD) is a disease with high mortality. We sought to develop an effective and convenient prediction tool to estimate mortality risk in patients with anti-MDA5 DM-ILD and inform clinical decision-making early.

**Methods:**

This prognostic study included Asian patients with anti-MDA5 DM-ILD hospitalized at the Nanjing Drum Hospital from December 2016 to December 2020. Candidate laboratory indicators were retrospectively collected. Patients hospitalized from 2016 to 2018 were used as the discovery cohort and applied to identify the optimal predictive features using a least absolute shrinkage and selection operator (LASSO) logistic regression model. A risk score was determined based on these features and used to construct the mortality risk prediction model in combination with clinical characteristics. Results were verified in a temporal validation comprising patients treated between 2019 and 2020. The primary outcome was mortality risk within one year. The secondary outcome was overall survival. The prediction model’s performance was assessed in terms of discrimination, calibration, and clinical usefulness.

**Results:**

This study included 127 patients, (72 men [56.7%]; median age, 54 years [interquartile range, 48-63 years], split into discovery (n = 87, 70%) and temporal validation (n=37, 30%) cohorts. Five optimal features were selected by LASSO logistic regression in the discovery cohort (n = 87) and used to construct a risk score, including lymphocyte counts, CD3+CD4+ T-cell counts, cytokeratin 19 fragment (CYFRA21-1), oxygenation index, and anti-Ro52 antibody. The retained predictive variables in the final prediction model were age, Heliotrope, fever, and risk score, and the most predictive factor was the risk score. The prediction model showed good discrimination (AUC: 0.915, 95% CI: 0.846–0.957), good calibration (Hosmer–Lemeshow test, P = 0.506; Brier score, 0.12), and fair clinical usefulness in the discovery cohort. The results were verified among patients in the temporal validation cohort (n = 38). We successfully divided patients into three risk groups with very different mortality rates according to the predictive score in both the discovery and validation cohorts (Cochran-Armitage test for trend, P < 0.001).

**Conclusions:**

We developed and validated a mortality risk prediction tool with good discrimination and calibration for Asian patients with anti-MDA5 DM-ILD. This tool can offer individualized mortality risk estimation and inform clinical decision-making.

## Introduction

Dermatomyositis (DM), as one idiopathic inflammatory myopathy (IIM), is an idiopathic inflammatory disease with inflammatory, immune-mediated organ damage. This disease can affect multiple organs, including the lung, muscle, skin, joints, and heart (1, 2). Interstitial lung disease (ILD) is the most common and severe complication of DM, contributing significantly to mortality (3, 4). Myositis-specific antibodies (MSAs), classical autoantibodies found in IIM patients, have been associated with specific clinical manifestations, disease progression, and treatment response (5–7). Among the MSAs, anti-melanoma differentiation-associated gene 5 (MDA5) autoantibodies are a unique subtype (8). The clinical manifestations, treatment response, and prognosis are highly heterogeneous among anti-MDA5 DM-ILD patients.

Rapid progression interstitial lung disease (RPILD), as a typical manifestation, is characterized by progressive deterioration of dyspnea and hypoxemia within three months. Despite immediately receiving an aggressive combined treatment of corticosteroids and immunosuppressive agents, more than 50% of anti-MDA5 DM-RPILD patients still experience a fatal outcome in the disease course due to resistance to the treatment (9, 10). In comparison, non-RPILD progresses slowly and responds favorably to conventional therapy (11–13). Accordingly, early prediction of the mortality risk of patients with anti-MDA5 DM-ILD remains challenging in clinical practice. Its accurate prediction is essential in informing clinical decision-making. The discovery of a convenient model useful for prediction could be driven to the removal of less-necessary clinical examinations, saving time and money, and reducing the burden on the patients (14–16).

Several potential parameters for distinguishing anti-MDA5 DM RPILD from anti-MDA5 DM non-RPILD and predicting survival in anti-MDA5 DM patients have been found, including demographics, imaging features, and laboratory indicators (17–21). Among them, laboratory indicators have the advantages of convenient use, objectivity, and minimal invasiveness. These biomarkers are not only correlated with disease activity but are also closely involved in the prognosis and therapeutic response of the disease. In anti-MDA5 DM-ILD patients, anti-Ro52 antibodies as myositis-associated autoantibodies (MAAs) antibodies often co-occur with anti-MDA5 antibodies. A combination of anti-Ro52 antibody status and anti-MDA5 antibody could help predict patients’ prognoses (22). Other circulating biomarkers reported to be associated with rapidly progressive ILD with high mortality include Krebs von den Lungen-6 (KL-6) (22), ferritin (21), macrophage-mannose receptor CD206 (19), and Cytokeratin 19 fragment (CYFRA21-1) (23). The limitation of these studies was that they investigated these biomarkers independently. A single biomarker cannot sufficiently predict the treatment response and mortality risk due to insufficient sample size and heterogenous cutoff values of biomarkers across these studies, which may result in inconsistent findings. A combination of these laboratory indicators could be a promising strategy. However, no study has investigated models incorporating multiple biomarker findings into clinical decision-making. Therefore, the current study sought to develop a pre-therapeutic prediction tool based on baseline clinical and laboratory indicators to predict mortality risk in Asian patients with anti-MDA5 DM-ILD and guide clinical decision-making early.

## Methods

### Study population

Patients with anti-MDA5 DM-ILD hospitalized at the Department of Respiratory and Critical Care Medicine in Nanjing Drum Tower Hospital between December 2016 and December 2020 were screened. Patients with overlapping syndromes or malignant tumors, such as systemic sclerosis, Sjogren’s syndrome, and lung cancer, were excluded. All the patients were aged above 18 years and had a follow-up period of at least 12 months. The Ethics Committee of the Nanjing Drum Tower Hospital approved the study protocol. Written informed consent was waived due to the nature of the retrospective study and anonymous processing of individual data. This retrospective prognostic study was reported following the Transparent Reporting of a Multivariable Prediction Model for Individual Prognosis or Diagnosis (TRIPOD) reporting guideline and conducted under the Declaration of Helsinki.

### Diagnostic criteria of anti-MDA5 DM-ILD

DM was diagnosed according to the Bohan and Peter criteria (1). The diagnosis of ILD was made according to respiratory symptoms (dry cough and dyspnea on exertion), physical examinations (Velcro rales), and abnormal chest high-resolution CT (HRCT) imaging (consolidations, reticulations, honeycombs, etc.), with the exclusion of infection, exposure to the environment and drug-induced interstitial lung disease. RP-ILD was defined as progressive dyspnea and a new appearance of interstitial abnormalities on HRCT within one month, excluding cardiac failure or fluid overload (24). Deterioration of ILD was diagnosed according to the revised diagnostic criteria described by Collard et al. in 2016 (25). Two expert radiologists independently evaluated the presence of ILD.

### Clinical and laboratory data

Clinical data for all patients were extracted from reviewing the medical records. Demographic information, such as the age of onset, sex, and clinical manifestations, including heliotrope sign, Gottron’s sign, Mechanic’s hands, Skin ulceration, Arthralgia, muscle weakness, and fever was obtained. The survival data were collected by reviewing the medical records or over telephone calls. Self-reported clinical manifestations were also recorded if they did complain at the follow-up.

This study detected MSAs and MAAs in patients using an immunoblotting method (EUROLINE, EUROIMMUN AG, Germany) by the same central lab while patients were first admitted to the hospital. Laboratory indicators were collected, including routine blood tests (WBC, lymphocyte, platelet), myositis antibodies, C-reactive protein (CRP), creatine kinase (CK), lactic dehydrogenase (LDH), and arterial blood gas analysis (PaO2, oxygenation index [OI]). If available, baseline pulmonary function testing parameters were recorded, including forced vital capacity predicted (FVC%) and diffusing capacity of the lung for carbon monoxide predicted (DLCO%).

Chest HRCT was performed in 127 patients at admission. Imaging appearances are mainly described as organizing pneumonia (OP) patterns and nonspecific interstitial pneumonia (NSIP) patterns combined with OP. Variables with missing values included CYFRA21-1 (n=5) and OI (n=1). The missing patterns are presented in **Figure S1**.

### Statistical analysis

The primary outcome was mortality risk within one year. The secondary outcome was overall survival. Continuous variables are presented as medians and interquartile ranges (IQRs) and were compared using the Mann–Whitney U test. Categorical variables were described as frequencies and percentages and compared using the χ2 test or Fisher’s exact test. Multiple imputations of the missing values were performed using a multivariable imputation by chained equations algorithm. Time-to-event data were estimated using the Kaplan–Meier method. We developed the prediction tool using a discovery cohort (n = 89) comprising patients with anti-MDA5 DM-ILD who underwent treatment between December 2016 and December 2018. The prediction tool was developed in two steps.

In the first step, a least absolute shrinkage and selection operator (LASSO) logistic regression model, along with 10-fold cross-validation, was applied to select the optimal predictive features among the candidate blood indicators in the discovery cohort. A risk score was subsequently constructed based on the corresponding coefficients of optimal features identified in the LASSO logistic regression using lambda.1 se. In the second step, the risk score and clinical characteristics were included in a stepwise multivariate logistic regression model. Model selection was based on the likelihood ratio test with Akaike’s information criterion (AIC) as the stopping rule. The model with the smallest AIC was selected as the optimal model and was further applied to develop the final prediction tool. The performance of the prediction tool was assessed for discrimination, calibration, and clinical usefulness. The discriminative ability was measured using the area under the receiver operating characteristic (AUC) curves. Bootstrapping with 2000 replicates was performed to generate the AUCs and corresponding 95% confidence intervals (CIs). Calibration was examined by the Hosmer–Lemeshow goodness-of-fit test, Brier score, and observed versus predicted graphs. Clinical usefulness was assessed using decision curve analysis (DCA). Data from an independent cohort of patients with anti-MDA5 DM-ILD who underwent treatment from January 2019 to December 2020 were used for temporal validation.

All statistical analyses were performed in R version 4.2.0 (R Foundation for statistical computing, Vienna, Austria). A two-sided P value of less than 0.05 was considered significant.

## Results

### Patient characteristics

This study included a total of 127 Asian patients (72 men [56.7%]; median age, 54 years [IQR, 48-63 years], divided into discovery (n = 89 [70%], diagnosed from 2016 to 2018) and temporal validation (38 [30%], diagnosed from 2019 to 2020) cohorts based on year of diagnosis. Descriptive statistics of patients in the discovery and temporal validation cohorts are summarized in **Table 1**. Patient characteristics were fairly comparable between the two groups. A summary of therapies administered to these patients is shown in **Table S1**. All patients had received corticosteroid as primary treatment. Among them, most patients (119 of 128, 92.9%) had received at least one line of immunosuppressive drug in addition to steroids, including cyclophosphamide (58.3%), tacrolimus (40.9%), tofacitinib (30.7%), and cyclosporin (11.0%). We also summarized the clinical characteristics of survivors and non-survivors in the discovery and validation cohorts in **Table S2**. There were 44 (49.4%) and 22 (57.9%) death events occurring within one year in the discovery cohort and validation cohort, respectively.

**Table 1:** Patient characteristics and treatments in discovery and temporal validation cohorts.

	All	Discovery cohort	Validation cohort	
Variable	(N = 127)	(N = 89)	(N = 38)	P value
Age, years	54.0 [48.0-63.0]	53.0 [47.0-63.0]	55.5 [51.2-66.0]	0.110
Gender				0.986
male	72 (56.7%)	51 (57.3%)	21 (55.3%)	
female	55 (43.3%)	38 (42.7%)	17 (44.7%)	
CT				0.729
NSIP/NSIP+OP	26 (20.5%)	17 (19.1%)	9 (23.7%)	
OP	101 (79.5%)	72 (80.9%)	29 (76.3%)	
Heliotrope				0.426
Absent	82 (64.6%)	55 (61.8%)	27 (71.1%)	
Present	45 (35.4%)	34 (38.2%)	11 (28.9%)	
Gottron sign^a^				0.855
Absent	37 (29.1%)	25 (28.1%)	12 (31.6%)	
Present	90 (70.9%)	64 (71.9%)	26 (68.4%)	
Mechanic’s hands				0.503
Absent	43 (33.9%)	28 (31.5%)	15 (39.5%)	
Present	84 (66.1%)	61 (68.5%)	23 (60.5%)	
Skin ulceration				0.992
Absent	102 (80.3%)	72 (80.9%)	30 (78.9%)	
Present	25 (19.7%)	17 (19.1%)	8 (21.1%)	
Arthralgia				1.000
Absent	102 (80.3%)	71 (79.8%)	31 (81.6%)	
Present	25 (19.7%)	18 (20.2%)	7 (18.4%)	
Muscle weakness^b^ dwwwweakness				0.579
Absent	105 (82.7%)	72 (80.9%)	33 (86.8%)	
Present	22 (17.3%)	17 (19.1%)	5 (13.2%)	
Fever				0.519
Absent	74 (58.3%)	54 (60.7%)	20 (52.6%)	
Present	53 (41.7%)	35 (39.3%)	18 (47.4%)	
Smoking status^c^				0.351
Absent	101 (79.5%)	70 (78.7%)	31 (81.6%)	
Present	26 (20.5%)	19 (21.3%)	7 (18.4%)	
WBC	6.80 [4.80-9.60]	6.70 [4.80-8.50]	6.80 [4.68-9.67]	0.784
PLT	193 [155-258]	193 [154-258]	190 [158-258]	0.960
lymphocyte	0.80 [0.57-1.20]	0.80 [0.60-1.20]	0.70 [0.50-0.90]	0.073
CD3^+^CD4^+^T	241 [150-408]	230 [140-430]	280 [188-375]	0.463
Ro52				0.730
Positive	79 (62.2%)	54 (60.7%)	25 (65.8%)	
Negative	48 (37.8%)	35 (39.3%)	13 (34.2%)	
CK	52.0 [30.0-114]	50.0 [29.0-105]	55.5 [32.0-114]	0.591
LDH	369 [271-496]	358 [267-502]	372 [274-459]	0.595
CRP	11.0 [4.70-35.2]	15.0 [4.70-35.7]	8.85 [4.50-24.2]	0.402
IgG	11.3 [9.15-13.4]	11.3 [9.50-13.6]	11.1 [8.90-12.5]	0.358
CYFRA211	6.76 [4.28-12.9]	6.89 [4.23-13.1]	5.96 [4.32-12.6]	0.653
OI	215 [158-304]	209 [146-310]	233 [196-298]	0.345

CT, computed tomography; NSIP, nonspecific interstitial pneumonia; OP, organizing pneumonia; WBC, white blood counts; PLT, platelets; CK, creatine kinase; LDH, lactate dehydrogenase; CRP, C-reactive protein; IgG, immunoglobulin G; CYFRA211, cytokeratin 19 fragment; OI, oxygenation index.

**^a^** Gottron’s sign and inverse Gottron’s sign were pooled in data collection.

**^b^** Muscle weakness was self-reported, referring to the decline of muscle function of the proximal extremities, manifested as arm lifting and hand lifting difficulties.

**^c^** The present category of smoking status only included the smoking status at presentation.

### Construction of the risk score

**Figure 1:**
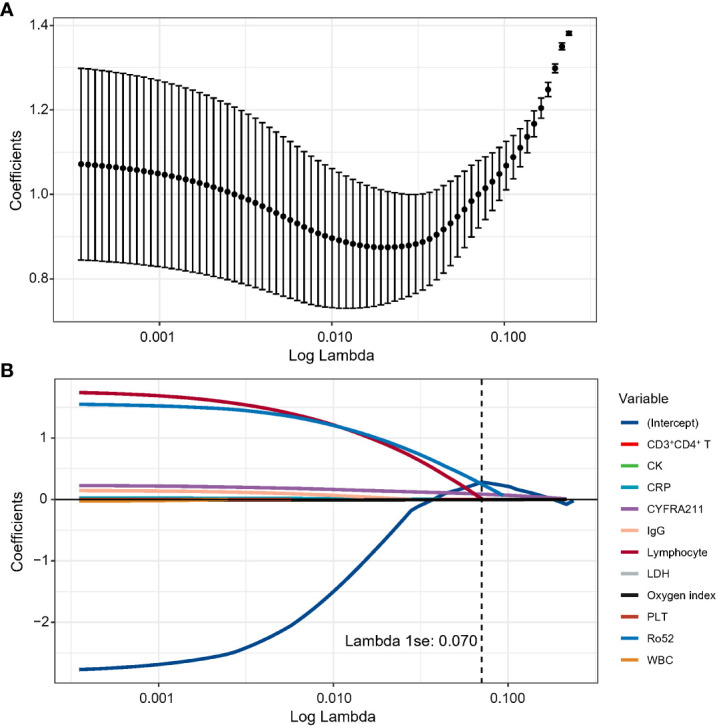
The optimal features were selected using a LASSO logistics regression. **(A)** Tuning parameter lambda selection in the LASSO method using 10-fold cross-validation *via* lambda.1se criteria in the discovery cohort. **(B)** LASSO coefficient profiles of the candidate blood indicators.

Eleven candidate laboratory indicators were reduced to the five most useful predictive markers using LASSO logistic regression, including lymphocytes, CD3^+^CD4^+^ T cells, cytokeratin 19 fragment (CYFRA211), oxygenation index (OI), and anti-Ro52 antibody (**Figure 1**). The risk score for each patient was calculated based on the values of selected features and corresponding coefficients derived from the LASSO logistic regression model. The calculation formula of the risk score was as follows: lymphocyte × 0.0057 - CD3^+^CD4^+^ T-cell × 0.00038 + Ro52 × 0.25 + CYFRA211 × 0.086 – OI × 0.0051.

### Development of the prediction tool

**Figure 2:**
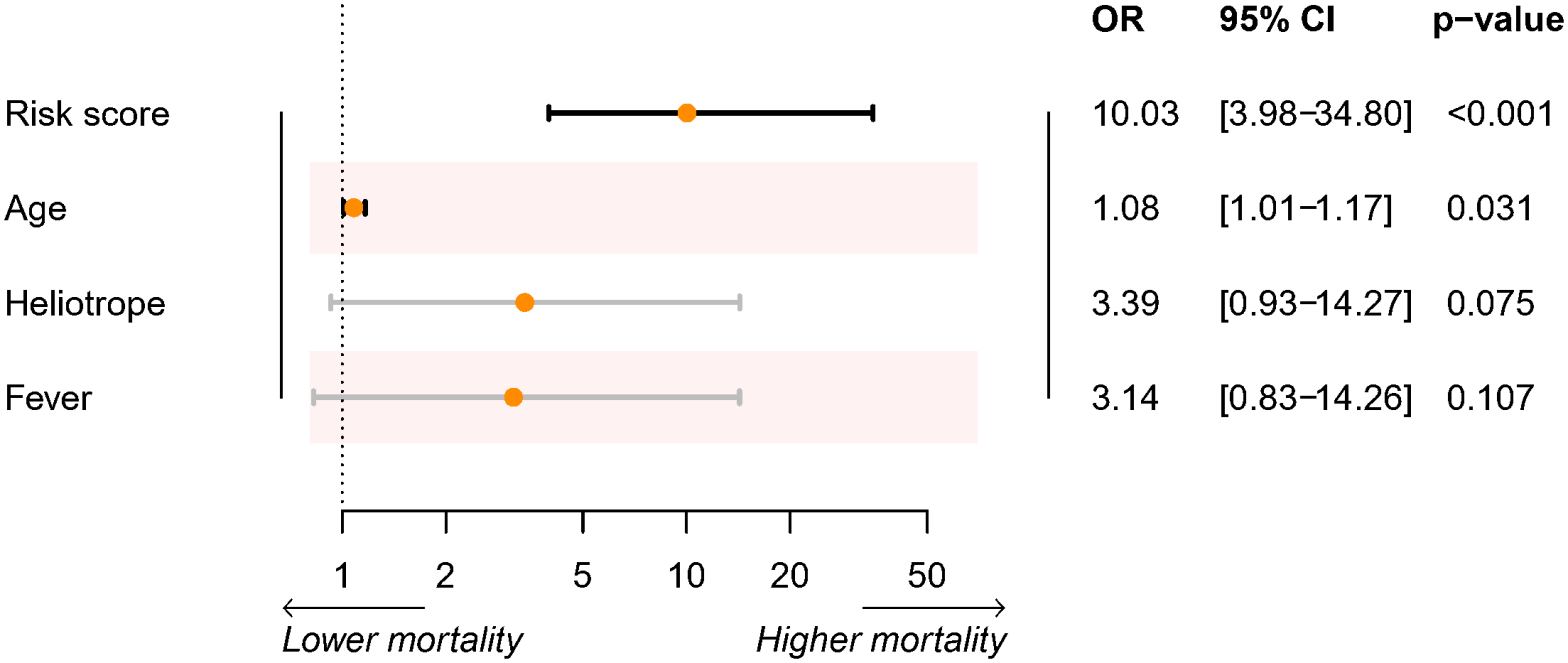
Summary of the optimal logistic regression model selected by the stepwise multivariable logistics regression model.

**Figure 3:**
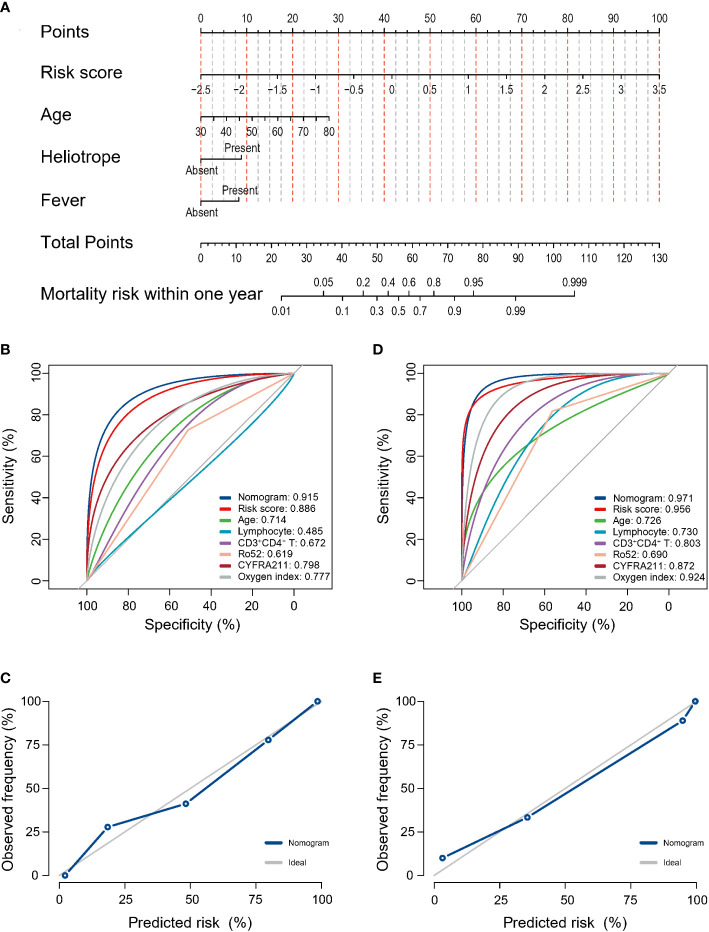
The prediction model of mortality risk for patients with anti-melanoma differentiation-associated gene 5 antibody-positive dermatomyositis with interstitial lung disease was visualized as a nomogram **(A)**. The discrimination was assessed by the area under the receiver operating characteristic (AUC) curves for the prediction model and other indicators in the discovery cohort **(B)** and temporal validation cohort **(C)**. Calibration was assessed using observed vs. predicted graphs in the discovery cohort **(D)** and temporal validation cohort **(E)**.

In the discovery cohort, the risk score was identified as a significant risk factor (OR: 9.27, 95% CI: 3.71-23.17; P < 0.001) in the univariate logistic regression analysis. In the stepwise multivariate modeling, we identified the optimal model with the lowest AIC of 72.53. The risk score was an important independent predictor (OR: 10.03, 95% CI: 3.98-34.80; P < 0.001). Other variables, including age, Heliotrope, and fever, were also retained in the optimal model (**Figure 2**). We established a prediction tool based on these factors and visualized it as a nomogram (**Figure 3A**). We also provided an online calculator for convenient use, which can be accessed through the website of https://shuangyiliu.shinyapps.io/DMwithILD/.

### Model performance assessment

**Figure 4:**
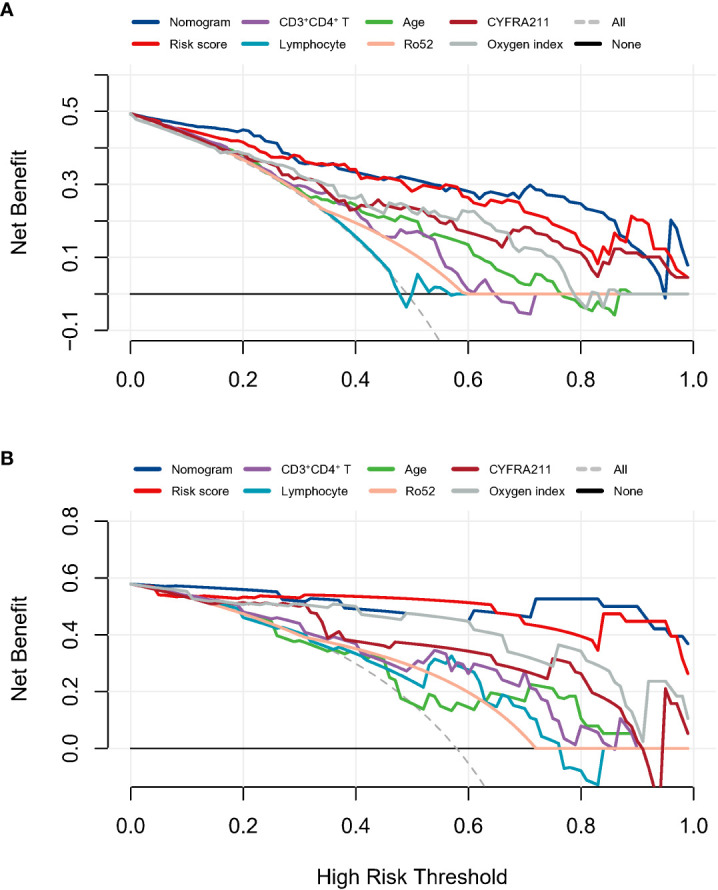
The decision curves analysis in the discovery cohort **(A)** and temporal validation cohort **(B)** assessed the clinical usefulness of the prediction model and other indicators.

The prediction tool showed good discrimination in the discovery cohort, with an AUC of 0.915 (95% CI: 0.846–0.957, **Figure 3B**), which was significantly higher than other variables (all P < 0.05) except for the risk score. Calibration was acceptable (Hosmer–Lemeshow test, P = 0.506; Brier score, 0.12; observed vs. predicted graphs, **Figure 3C**). DCA indicated that the prediction tool conferred more net benefits than the treat-all-patients scheme and the treat-no-patients scheme across all threshold probabilities (**Figure 4A**). The temporal validation cohort provided independent confirmation that the prediction tool had good discrimination (AUC: 0.971; 95% CI: 0.813–0.978, **Figure 3C**), well calibration (Hosmer-Lemeshow test, P = 0.390; Brier score, 0.06; observed vs. predicted graphs, **Figures 3D, E**), and clinical usefulness (**Figure 4B**).

### Risk stratification and clinical implication

**Figure 5:**
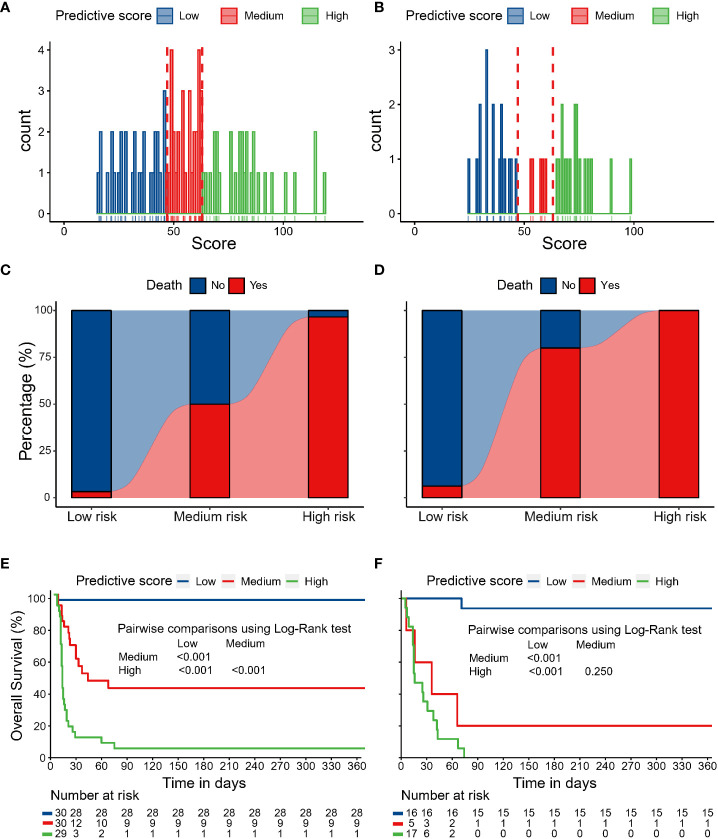
The distribution of nomogram scores in the discovery cohort **(A)** and temporal validation cohort **(B)**. Patients with higher nomogram scores were associated with higher mortality risk within one year in the discovery cohort **(C)** and temporal validation cohort **(D)**. Survival analyses revealed that patients in different risk groups had distinct survival probabilities at one year in the discovery cohort **(E)** and temporal validation cohort **(F)**.

The distribution of the nomogram score in the discovery and validation cohorts is shown in **Figures 5A, B**. Patients were categorized into three risk groups with different mortality rates based on the trisection values of the nomogram scores determined in the discovery cohort. Patients with a higher nomogram score were associated with a higher mortality risk within one year both in the discovery cohort (low risk: 3.3%, medium risk: 50%, high risk: 96.6%; Cochran-Armitage test for trend: P < 0.001; **Figure 5C**) and temporal validation cohort (low risk: 6.2%, medium risk: 80.0%, high risk: 100%; Cochran-Armitage test for trend: P < 0.001; **Figure 5D**). Survival analyses revealed that patients in different risk groups had distinct survival probabilities at one year in the discovery cohort (low vs. medium vs. high: 96.7% vs. 41.3% vs. 0%; **Figure 5E**) and temporal validation cohort (low vs. medium vs. high: 93.8% vs. 20.0% vs. 0%; **Figure 5F**).

We further explored the potential benefit of combined immunosuppressive drugs in those with higher risk according to our model. Given the limited sample size in this study, here we categorized patients into low- and high- risk groups based on median risk score to perform exploratory survival analysis. The analysis showed that high-risk patients had a tendency to benefit from the administration of two or more immunosuppressive drugs (log-rank P = 0.098, **Figure S2**), while low-risk patients showed not benefit (log-rank P = 0.490).

## Discussion

ILD is a severe complication of DM patients and is a leading contributor to poor prognosis, especially in anti-MDA5 DM-ILD (26–28). Here, based on a relatively large cohort of this rare disease, we successfully proposed an evidence-based prediction model for predicting mortality risk in Asian patients with anti-MDA5 DM-ILD based on clinical and laboratory indicators measured at diagnosis. We demonstrated that this prediction model had a good predictive ability when applied to a single institution using two different datasets. To the best of our knowledge, this was the first prediction tool available to predict individual mortality risk with a specific score for each anti-MDA5 DM-ILD patient. This tool has the potential to guide individualized treatment and reduce the risk of death by correctly identifying patients with anti-MDA5 DM-ILD at the highest mortality risk.

Our results indicated that death of the disease would mainly occur within 90 days from the onset of ILD, mainly in patients in the high-risk group. After 90 days, overall survival stabilized. The leading cause of death was related to disease progression in the 90 days. Notably, MDA5, an RNA-specific helicase, was an essential target of myositis-specific antibodies found in 10-35% of DM patients (29). Previous studies have suggested that MDA5 is a crucial antiviral factor that has been reported to be involved in viral pneumonia (30). High levels of anti-MDA5 Ab are closely associated with the severity and mortality of COVID-19 patients. MDA5 Ab-related hyper inflammation causes diffuse alveolar damage, resulting in acute respiratory distress syndrome (ARDS) (31). The death in anti-MDA5 DM-ILD could be accompanied by acute lung damage caused by the inflammatory factor storm.

In this study, higher serum LDH and CRP levels were observed in nonsurvivors. We presumed that elevated serum inflammatory cytokines (CRP and LDH) at admission were associated with acute epithelial cell damage and excessive inflammation. They might participate in disease progression, leading to death. In addition, CYFRA21-1 is a specific marker for lung cancer and is mainly expressed in alveolar and bronchial epithelial cells (32, 33). Dobashi N found markedly elevated serum CYFRA 21-1 levels are a meaningful indicator for monitoring acute epithelial cell damage in idiopathic pulmonary fibrosis (IPF) patients (34). Our previous study showed that elevated serum CYFRA 211 levels were associated with the severity and poor prognosis of ILD in anti-MDA5 DM patients (22). In this larger cohort, higher serum CYFRA 21-1 levels were also observed in nonsurvivors. Similar to a previous study, CYFRA 21-1 acted as an obvious risk factor for poor prognosis and was incorporated into the prediction model.

In the current study, the frequency of anti-Ro52 antibodies in anti-MDA5 DM-ILD patients was 74.7%. Anti-Ro52 antibodies, the most common myositis-associated autoantibodies (MAAs), often co-occur with anti-MDA5 antibodies. Anti-Ro52 antibodies coexisting with anti-MDA5 antibodies could more frequently cause disease progression, leading to unfavorable outcomes (21, 35). Our study also observed the high frequency of anti-Ro52 antibodies in anti-MDA5-DMILD. Consistent with a previous study, the existence of the anti-Ro52 antibody acted as an essential risk factor for a worse outcome, which was also incorporated into the model.

Clinically, lymphocyte-mediated immune mechanisms are considered to play an essential role in the pathogenesis of DM-ILD. A previous study by Sun et al. demonstrated that lymphocyte counts are much lower in CADM patients with acute/subacute ILD than in those with chronic ILD (36). Similarly, Chen et al. reported that the percentage of decreased CD4^+^ T-cell counts in peripheral blood is higher in anti-MDA5 DM-RPILD patients than in non-RPILD patients (37). To date, there have not been any studies about the clear pathophysiology between lymphocyte subgroups in peripheral blood and anti-MDA5 DM-ILD. Our results indicated a noticeable decrease in peripheral blood lymphocyte counts, mainly in CD3^+^CD4^+^ T cells, in nonsurvivors of anti-MDA5 DM-ILD patients compared with survivors. Our results showed that lymphocyte counts and CD3^+^CD4^+^ T-cell counts would help evaluate disease prognosis, despite the unclear pathophysiology, which was also incorporated into the prediction tool.

Moreover, pulmonary function testing was critical in assessing the disease severity. However, some patients could not complete lung function tests due to their serious condition at admission. Therefore, we chose OI as an index to indirectly evaluate patients’ pulmonary function, which indirectly demonstrated the diffusing capacity of the lung for carbon monoxide. Notably, some clinical manifestations, such as heliotrope rash and fever, were more frequently observed in nonsurvivors of anti-MDA5 DM-ILD patients. Hence, such clinical features were also included in developing the mortality risk prediction tool. Given the high mortality risk of anti-MDA5 DM-ILD, early evaluation of the mortality risk of this disease would be helpful once a patient is diagnosed. The prediction model we proposed could help us better understand the severity of the disease and guide clinical decision-making and individual treatment. Also, this tool can potentially assist clinical research design in MDA5 DM-ILD patients based on different mortality risks. In this study, the exploratory analysis showed that high-risk patients (scored top 50%) had a tendency to benefit from the administration of two or more immunosuppressive drugs, while low-risk patients showed not benefit. Nevertheless, the finding is a hypothesized one and could be affected by potential confounding factors. A large prospective cohort is needed to examine the finding and to draw more reliable conclusions.

Our study has several limitations. First, our ability to perform serial analysis of clinical parameters was limited because of the lack of data, such as lung function, which was understandable given the severity of anti-MDA5 DM-ILD. Therefore, we applied the oxygenation index as a surrogate indicator. Though not perfect, it may partially reflect the patient’s lung function. Second, this was a retrospective, single-institution study. All patients were Asians. Further multicenter external validation, especially in other racial populations, should be performed to verify our prediction model’s predictive ability and generalizability in future studies. Third, all patients in the study were Asians, the algorithm is only applicable at the moment to Asian populations. Lastly, artificial intelligence (AI) represents computer processes that allow performing complex data analyses with the least human intervention (15, 16). The use of AI in medicine significantly expanded recently. How to apply AI to analyze huge amounts of information and accordingly makes decisions warrants further investigation in this domain.

In conclusion, based on baseline clinical and laboratory indicators, we developed and validated a predictive tool with good predictive ability in Asian patients with anti-MDA5 DM-ILD. It accurately estimates individual mortality risk, which helps inform clinical decision-making early.

## Data availability statement

The raw data supporting the conclusions of this article will be made available by the authors, without undue reservation.

## Ethics statement

The studies involving human participants were reviewed and approved by The Ethics Committee of the Nanjing Drum Tower Hospital. Written informed consent for participation was not required for this study in accordance with the national legislation and the institutional requirements.

## Author contributions

YX and XG had full access to all the data in the study and took responsibility for the data’s integrity and the data analysis’s accuracy. XG, WL, and YY contributed equally to this study. Concept and design: YX, MH, and XG. Acquisition, analysis, or interpretation of data: All authors. Drafting of the manuscript: XG, WaL, and YY. Critical revision of the manuscript for important intellectual content: All authors. Statistical analysis: All authors. Obtained funding: YX, MH and XG. Administrative, technical, or material support: YX and MH. Supervision: YX, XG, MiC. All authors contributed to the article and approved the submitted version.

## Funding

This work was supported by the National Natural Science Foundation of China (Grant No. 82070064), the Special Fund for Clinical Research from the Affiliated Drum Tower Hospital of Nanjing University Medical School (2021-LCYJ-PY-05), and the Special Fund for Clinical Research (Single Disease Database) from the Affiliated Drum Tower Hospital of Nanjing University Medical School (2021-LCYJ-DBZ-06).

## Conflict of interest

The authors declare that the research was conducted in the absence of any commercial or financial relationships that could be construed as a potential conflict of interest.

## Publisher’s note

All claims expressed in this article are solely those of the authors and do not necessarily represent those of their affiliated organizations, or those of the publisher, the editors and the reviewers. Any product that may be evaluated in this article, or claim that may be made by its manufacturer, is not guaranteed or endorsed by the publisher.
