# Supplementary material for: Prediction model for the pretreatment evaluation of mortality risk in anti-melanoma differentiation-associated gene 5 antibody-positive dermatomyositis with interstitial lung disease
Source: Front Immunol. 2022 Sep 23;13:978708. doi: 10.3389/fimmu.2022.978708 (PMC9539924; doi:10.3389/fimmu.2022.978708)
Supplement: Supplementary file 1 [file DataSheet_1.docx]

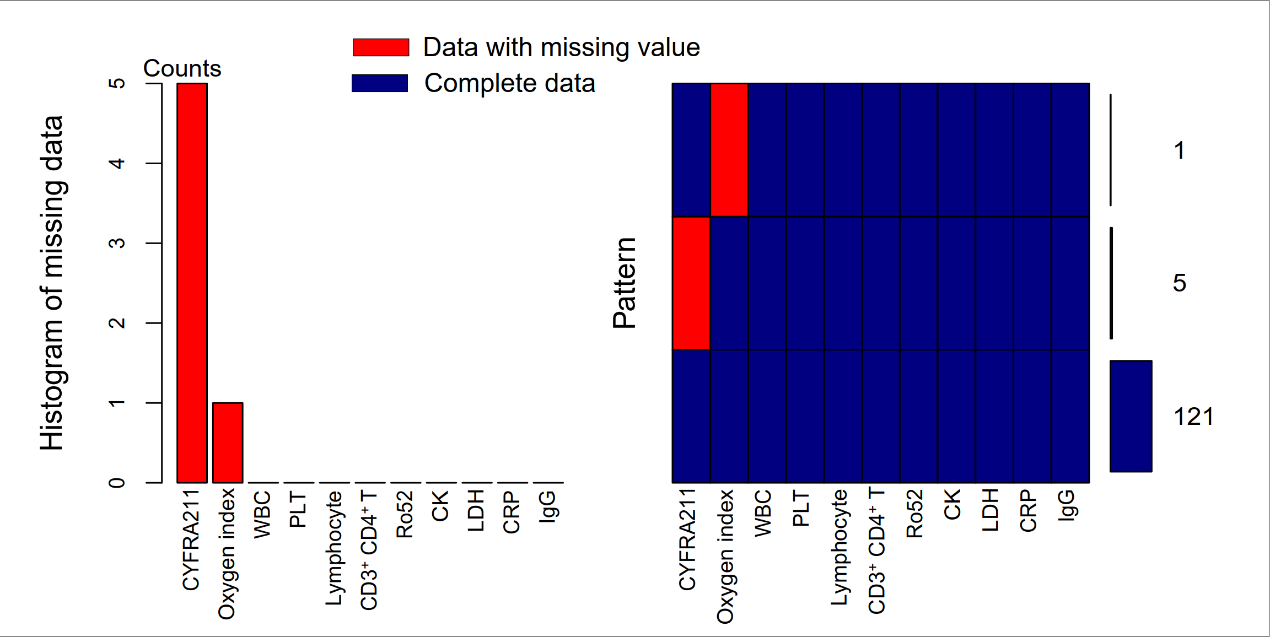


**Figure S1.** The missing patterns of variables with missing values.


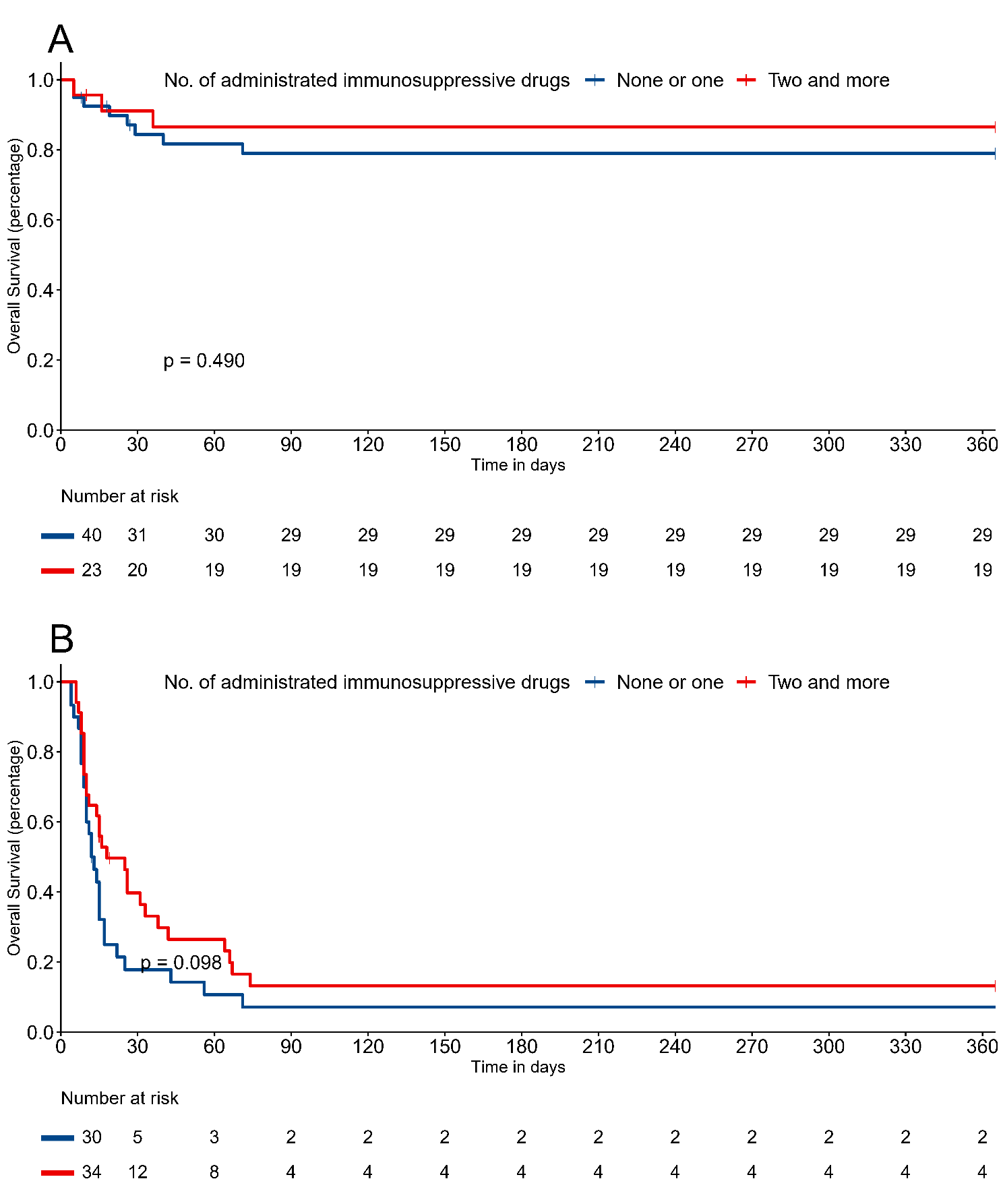


**Figure S2.** Exploratory analysis of the effect of number of administrated immunosuppressive drugs on survival in low- and high-risk patients. (Patients were categorized into low- and high- risk groups based on median risk score.)

| **Table S1.** Summary of therapies administrated to patients in discovery and temporal validation cohorts**.** | | | | |
| --- | --- | --- | --- | --- |
|  | **All** | **Discovery cohort** | **Validation cohort** |  |
| **Variable** | **(N = 127)** | **(N = 89)** | **(N = 38)** | **P value** |
| Corticosteroid: Yes | 127 (100%) | 89 (100%) | 38 (100%) | . |
| IVIG |  |  |  | 0.092 |
| No | 64 (50.4%) | 40 (44.9%) | 24 (63.2%) |  |
| Yes | 63 (49.6%) | 49 (55.1%) | 14 (36.8%) |  |
| IVCY |  |  |  | 0.004 |
| No | 53 (41.7%) | 45 (50.6%) | 8 (21.1%) |  |
| Yes | 74 (58.3%) | 44 (49.4%) | 30 (78.9%) |  |
| Tacrolimus |  |  |  | 0.981 |
| No | 75 (59.1%) | 52 (58.4%) | 23 (60.5%) |  |
| Yes | 52 (40.9%) | 37 (41.6%) | 15 (39.5%) |  |
| Cyclosporin |  |  |  | 0.227 |
| No | 113 (89.0%) | 77 (86.5%) | 36 (94.7%) |  |
| Yes | 14 (11.0%) | 12 (13.5%) | 2 (5.26%) |  |
| Tofacitinib |  |  |  | 0.001 |
| No | 88 (69.3%) | 70 (78.7%) | 18 (47.4%) |  |
| Yes | 39 (30.7%) | 19 (21.3%) | 20 (52.6%) |  |
| **Abbreviation:** IVIG, intravenous immunoglobulin; IVCY, intravenous cyclophosphamide. | | | | |

| **Table S2.** Clinical characteristics in survivors and non-survivors in discovery and temporal validation cohorts. | | | | | | | | |
| --- | --- | --- | --- | --- | --- | --- | --- | --- |
|  |  | **Discovery cohort** | | |  | **Validation cohort** | | |
|  |  | **Survivor** | **Non-survivor** |  |  | **Survivor** | **Non-survivor** |  |
| **Characteristics** |  | **(N = 45)** | **(N = 44)** | **P value** |  | **(N = 16)** | **(N = 22)** | **P value** |
| Age, years |  | 50.0 [45.0-54.0] | 59.5 [51.0-65.0] | <0.001 |  | 54.0 [47.8-58.2] | 62.0 [52.0-67.0] | 0.023 |
| Gender |  |  |  | 0.463 |  |  |  | 0.273 |
| Male |  | 28 (62.2%) | 23 (52.3%) |  |  | 11 (68.8%) | 10 (45.5%) |  |
| Female |  | 17 (37.8%) | 21 (47.7%) |  |  | 5 (31.2%) | 12 (54.5%) |  |
| CT |  |  |  | 0.258 |  |  |  | 1.000 |
| NSIP/NSIP+OP |  | 6 (13.3%) | 11 (25.0%) |  |  | 4 (25.0%) | 5 (22.7%) |  |
| OP |  | 39 (86.7%) | 33 (75.0%) |  |  | 12 (75.0%) | 17 (77.3%) |  |
| Heliotrope |  |  |  | 0.041 |  |  |  | 1.000 |
| Absent |  | 33 (73.3%) | 22 (50.0%) |  |  | 11 (68.8%) | 16 (72.7%) |  |
| Present |  | 12 (26.7%) | 22 (50.0%) |  |  | 5 (31.2%) | 6 (27.3%) |  |
| Gottron’s sign^a^ |  |  |  | 0.177 |  |  |  | 1.000 |
| Absent |  | 16 (35.6%) | 9 (20.5%) |  |  | 5 (31.2%) | 7 (31.8%) |  |
| Present |  | 29 (64.4%) | 35 (79.5%) |  |  | 11 (68.8%) | 15 (68.2%) |  |
| Mechanic’s hands |  |  |  | 0.876 |  |  |  | 1.000 |
| Absent |  | 15 (33.3%) | 13 (29.5%) |  |  | 6 (37.5%) | 9 (40.9%) |  |
| Present |  | 30 (66.7%) | 31 (70.5%) |  |  | 10 (62.5%) | 13 (59.1%) |  |
| Skin ulceration |  |  |  | 0.876 |  |  |  | 1.000 |
| Absent |  | 15 (33.3%) | 13 (29.5%) |  |  | 6 (37.5%) | 9 (40.9%) |  |
| Present |  | 30 (66.7%) | 31 (70.5%) |  |  | 10 (62.5%) | 13 (59.1%) |  |
| Arthralgia |  |  |  | 0.205 |  |  |  | 1.000 |
| Absent |  | 33 (73.3%) | 38 (86.4%) |  |  | 13 (81.2%) | 18 (81.8%) |  |
| Present |  | 12 (26.7%) | 6 (13.6%) |  |  | 3 (18.8%) | 4 (18.2%) |  |
| Muscle weakness^b^ |  |  |  | 1.000 |  |  |  | 1.000 |
| Absent |  | 36 (80.0%) | 36 (81.8%) |  |  | 14 (87.5%) | 19 (86.4%) |  |
| Present |  | 9 (20.0%) | 8 (18.2%) |  |  | 2 (12.5%) | 3 (13.6%) |  |
| Fever |  |  |  | 0.069 |  |  |  | 0.007 |
| Absent |  | 32 (71.1%) | 22 (50.0%) |  |  | 13 (81.2%) | 7 (31.8%) |  |
| Present |  | 13 (28.9%) | 22 (50.0%) |  |  | 3 (18.8%) | 15 (68.2%) |  |
| Smoking^c^ |  |  |  | 1.000 |  |  |  | 0.675 |
| Absent |  | 35 (77.8%) | 35 (79.5%) |  |  | 14 (87.5%) | 17 (77.3%) |  |
| Present |  | 10 (22.2%) | 9 (20.5%) |  |  | 2 (12.5%) | 5 (22.7%) |  |
| WBC |  | 5.80 [4.80-7.50] | 7.90 [4.97-10.5] | 0.079 |  | 5.80 [4.47-9.33] | 8.95 [5.32-10.2] | 0.124 |
| PLT |  | 223 [165-282] | 184 [150-244] | 0.125 |  | 197 [166-231] | 180 [147-295] | 0.836 |
| Lymphocyte |  | 0.90 [0.50-1.20] | 0.80 [0.60-1.25] | 0.951 |  | 0.90 [0.69-1.22] | 0.61 [0.40-0.71] | 0.011 |
| CD3^+^CD4^+^T |  | 330 [180-580] | 190 [122-258] | 0.003 |  | 340 [302-517] | 216 [134-287] | 0.001 |
| Ro52 |  |  |  | 0.037 |  |  |  | 0.036 |
| Positive |  | 22(48.9%) | 32(72.7%) |  |  | 7(43.8%) | 18(81.8%) |  |
| Negative |  | 23(51.1%) | 12(27.3%) |  |  | 9(56.2%) | 4(18.2%) |  |
| CK |  | 46.0 [28.0-90.0] | 53.5 [31.2-124] | 0.470 |  | 48.5 [30.0-60.5] | 65.0 [41.0-155] | 0.110 |
| LDH |  | 280 [234-358] | 462 [365-620] | <0.001 |  | 271 [220-310] | 448 [370-582] | <0.001 |
| CRP |  | 7.50 [4.60-20.0] | 21.8 [7.68-39.7] | 0.014 |  | 6.20 [3.32-8.62] | 21.0 [7.37-50.8] | 0.005 |
| IgG |  | 11.3 [9.20-13.6] | 11.1 [9.57-13.6] | 0.964 |  | 10.6 [8.65-11.7] | 11.2 [9.32-13.2] | 0.329 |
| CYFRA211 |  | 4.63 [3.12-7.40] | 12.4 [6.28-17.3] | <0.001 |  | 3.55 [2.50-5.16] | 11.6 [6.41-15.1] | <0.001 |
| OI |  | 266 [212-376] | 158 [93.5-209] | <0.001 |  | 305 [277-353] | 198 [154-218] | <0.001 |
| **Abbreviation:** CT, computed tomography; NSIP, nonspecific interstitial pneumonia; OP, organizing pneumonia; WBC, white blood counts; PLT, platelets; CK, creatine kinase; LDH, lactate dehydrogenase; CRP, C-reactive protein; IgG, immunoglobulin G; CYFRA211, cytokeratin 19 fragment; OI, oxygenation index.  ^a^: Gottron's sign and inverse Gottron's sign were pooled in data collection.  **^b^:** Muscle weakness was self-reported, referring to the decline of muscle function of the proximal extremities, manifested as arm lifting and hand lifting difficulties.  **^c^:** The present category of smoking status only included the smoking status at presentation. | | | | | | | | |

| **Table S3.** Therapies administrated to survivors and non-survivors in discovery and temporal validation cohorts. | | | | | | | | |
| --- | --- | --- | --- | --- | --- | --- | --- | --- |
|  |  | **Discovery cohort** | | |  | **Validation cohort** | | |
|  |  | **Survivor** | **Non-survivor** |  |  | **Survivor** | **Non-survivor** |  |
| **Drugs** |  | **(N = 45)** | **(N = 44)** | **P value** |  | **(N = 16)** | **(N = 22)** | **P value** |
| Corticosteroid: Yes |  | 45 (100%) | 44 (100%) | - |  | 16 (100%) | 22 (100%) | - |
| IVIG |  |  |  | <0.001 |  |  |  | 0.003 |
| No |  | 29 (64.4%) | 11 (25.0%) |  |  | 15 (93.8%) | 9 (40.9%) |  |
| Yes |  | 16 (35.6%) | 33 (75.0%) |  |  | 1 (6.25%) | 13 (59.1%) |  |
| IVCY |  |  |  | 0.595 |  |  |  | 0.698 |
| No |  | 21 (46.7%) | 24 (54.5%) |  |  | 4 (25.0%) | 4 (18.2%) |  |
| Yes |  | 24 (53.3%) | 20 (45.5%) |  |  | 12 (75.0%) | 18 (81.8%) |  |
| Tacrolimus |  |  |  | 0.603 |  |  |  | 1.000 |
| No |  | 28 (62.2%) | 24 (54.5%) |  |  | 10 (62.5%) | 13 (59.1%) |  |
| Yes |  | 17 (37.8%) | 20 (45.5%) |  |  | 6 (37.5%) | 9 (40.9%) |  |
| Cyclosporin |  |  |  | 0.725 |  |  |  | 0.499 |
| No |  | 40 (88.9%) | 37 (84.1%) |  |  | 16 (100%) | 20 (90.9%) |  |
| Yes |  | 5 (11.1%) | 7 (15.9%) |  |  | 0 (0.00%) | 2 (9.09%) |  |
| Tofacitinib |  |  |  | 0.327 |  |  |  | 1.000 |
| No |  | 33 (73.3%) | 37 (84.1%) |  |  | 8 (50.0%) | 10 (45.5%) |  |
| Yes |  | 12 (26.7%) | 7 (15.9%) |  |  | 8 (50.0%) | 12 (54.5%) |  |
| **Abbreviation:** IVIG, intravenous immunoglobulin; IVCY, intravenous cyclophosphamide. | | | | | | | | |
